# Blood Pictures : An Introduction to Clinical Hæmatology

**Published:** 1919

**Authors:** 


**Blood Pictures: An Introduction to Clinical Hæmatology.** By Cecil Price-Jones, M.B. Pp. 91. Bristol: John Wright & Sons. 1917. Price 6s. 6d. net.

This is not a text-book of hæmatology, but rather a guide to the interpretation of the elaborate reports on blood examinations made by the pathologists, and is also intended to assist the general practitioner in making examinations for himself. The normal blood picture is first described, and the two principal varieties known as the leucoid and the lymphoid types follow. "Speaking broadly, a leucoid type is associated with a *cœcal* infection, a lymphoid type of blood with a *bacillary* infection, and a lymphoid plus large mononuclear type with a *protozoal* infection." These varieties are further explained in subsequent chapters. The characteristics of some varieties of Anæmia are then indicated, and the blood picture of malignant disease with its utility in the differentiation from non-malignant conditions is considered. An excellent coloured plate showing the phylogenetic relations of blood-cells concludes this very enlightening book, which should be studied by those who are not familiar with the larger text-books.

